# Tangeretin attenuates bleomycin-induced pulmonary fibrosis by inhibiting epithelial-mesenchymal transition via the PI3K/Akt pathway

**DOI:** 10.3389/fphar.2023.1247800

**Published:** 2023-09-15

**Authors:** Jiang Li, Qian Wei, Ke Song, Youxin Wang, Yuxin Yang, Miao Li, Jiaying Yu, Guangxu Su, Luyuan Peng, Bendong Fu, Pengfei Yi

**Affiliations:** ^1^ College of Veterinary Medicine, Jilin University, Changchun, China; ^2^ Department of Internal Medicine-Cardiovascular, The Third Affiliated Hospital of Changchun University of Traditional Chinese Medicine, Changchun, China

**Keywords:** pulmonary fibrosis, tangeretin, PI3K/Akt signaling pathway, epithelial-mesenchymal transition, bleomycin

## Abstract

**Background:** Pulmonary fibrosis (PF) is a terminal pathological change in a variety of lung diseases characterized by excessive deposition of extracellular matrix, for which effective treatment is lacking. Tangeretin (Tan), a flavonoid derived from citrus, has been shown to have a wide range of pharmacological effects. This study aimed to investigate the role and potential mechanisms of Tan on pulmonary fibrosis.

**Methods:** A model of pulmonary fibrosis was established by administering bleomycin through tracheal drip, followed by administering Tan or pirfenidone through gavage. HE and Masson staining were employed to assess the extent of pulmonary fibrosis. Subsequently, Western blot, enzyme-linked immunosorbent assay (ELISA), RNA sequencing, and immunohistochemistry techniques were employed to uncover the protective mechanism of Tan in PF mice. Furthermore, A549 cells were stimulated with TGF-β1 to induce epithelial-mesenchymal transition (EMT) and demonstrate the effectiveness of Tan in mitigating PF.

**Results:** Tan significantly ameliorated bleomycin-induced pulmonary fibrosis, improved fibrotic pathological changes, and collagen deposition in the lungs, and reduced lung inflammation and oxidative stress. The KEGG pathway enrichment analysis revealed a higher number of enriched genes in the PI3K/Akt pathway. Additionally, Tan can inhibit the EMT process related to pulmonary fibrosis.

**Conclusion:** Taken together, the above research results indicate that Tan suppresses inflammation, oxidative stress, and EMT in BLM-induced pulmonary fibrosis via the PI3K/Akt pathway and is a potential agent for the treatment of pulmonary fibrosis.

## 1 Introduction

Pulmonary fibrosis (PF) is a chronic and lethal lung disease that is characterized by the excessive deposition of extracellular matrix (ECM), resulting in impaired gas exchange and lung function (Moss et al., 2022; C; Wang and Yang, 2022; J; Yang et al., 2033a). The most prevalent form of PF is idiopathic pulmonary fibrosis, which has a bleak prognosis and a median survival rate of 2.5–3.5 years following diagnosis (Strongman et al., 2018). Pulmonary fibrosis is associated with multiple risk factors, such as genetic predisposition, exposure to radiation and environmental toxins, adverse drug reactions, and age and gender (Mathai and Schwartz, 2019). Additionally, various respiratory viruses, including the common influenza virus, severe acute respiratory syndrome coronavirus 2 (SARS-CoV-2), and respiratory syncytial virus, have been identified as potential causative agents of pulmonary fibrosis (Wang et al., 2017; Chiou et al., 2023; W; Yang et al., 2022). Currently, pulmonary fibrosis is treated with pirfenidone (PFD) and nintedanib, which have limited efficacy in halting the progression of fibrosis and are associated with varying degrees of adverse effects, thereby hindering their widespread use (Trachalaki et al., 2021). Consequently, novel therapeutic approaches are warranted for the prevention and management of pulmonary fibrosis.

The pathogenesis of pulmonary fibrosis remains uncertain; however, current consensus suggests that it initiates with anomalous tissue restoration following injury (Sun et al., 2023; H; Li, Zhao, Tian, et al., 2020). Following lung injury, alveolar type II (AT2) cells participate in the repair process through self-renewal and differentiation into alveolar type I epithelial cells (AT1)(L. Shao et al., 2021; P; Wang, Yan, et al., 2022; Tan et al., 2021). Furthermore, AT2 cells may acquire a mesenchymal phenotype via epithelial-mesenchymal transition (EMT), stimulate fibroblast activation and differentiation, and generate a substantial quantity of extracellular matrix (ECM) molecules implicated in pulmonary fibrosis (Olajuyin et al., 2019; Zhou et al., 2022). Transforming growth factor-β1 (TGF-β1) is acknowledged as a pro-fibrotic factor in various organs (Meng et al., 2016). Its mechanism of action involves binding to the TGF-β type II receptor on the AT2 cell membrane and recruiting the TGF-β type I receptor to the plasma membrane, resulting in the formation of a heterotrimer that activates the downstream Smads signaling pathway and the expression of EMT-related transcription factors Snail, Slug, and Twist1(P. Wang, Yan, et al., 2022; Garg, 2013). Additionally, TGF-β1 can induce EMT through a non-Smad-dependent pathway, whereby it stimulates the expression of the PI3K subunit p110 and phosphorylation of Akt to promote EMT (Y.E. Zhang, 2017; Saito et al., 2017). The activation of Akt is facilitated through the regulation of its downstream proteins, such as GSK-3β, mTOR, HIF-1α, and NF-κB, which are implicated in the pathogenesis of pulmonary fibrosis (Qin et al., 2021; Peng et al., 2022; Xu et al., 2022).

Tangeretin (Tan) is a naturally occurring flavonoid mainly found in the peel peel of citrus plants. It exhibits various pharmacological properties, such as antioxidant and anti-inflammatory effects (Xin, et al., 2019; Sedik and Elgohary, 2023).Tan is absorbed by the gastrointestinal tract and primarily metabolized to 4′-demethyltangeretin by CYP1A1 and CYP1A2 enzymes (Surichan et al., 2018). Tan has the potential to mitigate acute lung injury induced by LPS through the inhibition of the Th17 response, TNF-α, and MPO activity via the Notch signaling pathway (M. Li, Zhao, Qi, et al., 2020). Previous studies have demonstrated that Tanis able to prevent podocyte injury and renal fibrosis by effectively blocking glucose-induced oxidative stress and hypoxia-induced EMT in podocytes (Kang et al., 2020). In addition, Tan effectively suppressed the activation of JAK2/STAT3, Wnt, and EGFR signaling pathways, as well as lung fibroblast activation (F. Yang J. et al., 2023; D; Shao et al., 2022). However, the potential mechanism of Tan in the treatment of pulmonary fibrosis remains incompletely understood. In this study, we employed network pharmacology to predict the potential biological pathways of Tan in the management of pulmonary fibrosis. Additionally, we conducted experimental investigations to investigate the function of Tan in pulmonary fibrosis.

## 2 Materials and methods

### 2.1 Chemicals and reagents

Tan was purchased from Chengdu Must Biotechnology Co., Ltd. (purity≥98%, Chengdu, China). Bleomycin sulfate (BLM) was obtained from Macklin (Shanghai, China). Pirfenidone was purchased from Solarbio (Beijing, China). The primary antibodies against E-cadherin, N-cadherin, MMP9, and collagen I were purchased from Immunoway (Plano, TX, United States). p-PI3K, PI3K, p-Akt1, and Akt1 were from Affinity Bioscience (Changzhou, China). TGF-β1, TGFBR2, α-SMA, β-actin, and all the secondary antibodies were from the Proteintech Group (Wuhan, China).

### 2.2 Animal model and treatment

Thirty-six 6–8 weeks old male C57BL/6 mice (weight 18–22 g) were purchased from Liaoning Changsheng Biotechnology Co., Ltd. and randomly divided into 6 groups: control, BLM, BLM + Tan (10 mg/kg, Tan 10), BLM + Tan (20 mg/kg, Tan 20), BLM + Tan (40 mg/kg, Tan 40), and BLM + pirfenidone (PFD). The pulmonary fibrosis mice model was constructed by intra-tracheal drip with 5 mg/kg of BLM at day 0. The control mice received an equal amount of saline intravenously. The following day after BLM treatment by gavage, once daily for 21 days: the positive drug group was given pirfenidone by gavage (200 mg/kg/d), Tan powder dissolved in 0.5% sodium carboxymethylcellulose (CMC-Na) solution by gavage (10 mg/kg/d, 20 mg/kg/d, 40 mg/kg/d), and the control and BLM groups were given equal volumes of 0.5% CMC-Na. Body weights were measured every 7 days. On the last day, the mice were euthanized and lung tissues were collected for subsequent experiments. All experiments were conducted in strict accordance with the Jilin University Guide for the Use and Welfare of Laboratory Animals and were approved by the Jilin University Animal Testing Ethics Committee (approval number: SY202212001).

### 2.3 Cell culture and treatment

A549 cells were bought from the Cell Bank of the Chinese Academy of Sciences (Shanghai, China). Cells were cultured at 37°C under 5% CO_2_ in Dulbecco’s Modified Eagle Media (DMEM; HyClone) with 10% fetal bovine serum (Biological Industries) and 100 U/mL penicillin and 100 μg/mL streptomycin. The cytotoxicity of Tan to A549 cells was determined by the CCK-8 assay. An *in vitro* pulmonary fibrosis model was established using 5 ng/mL TGF-β1 stimulated A549 cells as described previously (Weng et al., 2018; W; Liu et al., 2022). Briefly, A549 cells were seeded into 6-well plates and starved for 24 h following co-culture for 24 h at different Tan concentrations with or without TGF-β1 (Peprotech, New Jersey, United States).

### 2.4 Histopathological analysis of lung tissues

The left lung was fixed in 4% paraformaldehyde for 24 h, embedded in paraffin, and cut to 4 µm. The sections were then stained with hematoxylin-eosin (H&E) and Masson’s trichrome stain. Fibrosis scoring and pulmonary fibrosis area assessment were performed as described previously (Hübner et al., 2008; C.-Y; Chen et al., 2010). Briefly, the area of blue collagen fibers in the whole Masson stained scanned section was quantified using ImageJ software in a 2 × field of view, with the degree of pulmonary fibrosis expressed as a percentage of collagen fiber area. The area fraction of fibrosis = collagen fiber area/lung tissue area × 100%.

### 2.5 Determination of hydroxyproline, oxidative stress, and inflammatory cytokines in lung tissues

The hydroxyproline, SOD, CAT, and MDA (Nanjing Jiancheng, Nanjing, China) contents in the mice lung tissues were detected according to the manufacturer’s instructions. The levels of IL-1β, IL-6, and TNF-α (BioLegend, United States) in lung tissue homogenates were measured using ELISA kits according to the manufacturer’s instructions.

### 2.6 Quantitative real-time polymerase chain reaction

Total RNA was extracted using TRIzol^®^ Reagent (Magen) and then reverse transcribed into cDNA using a TransScript^®^ Uni All-in-One First-Strand cDNA Synthesis SuperMix kit (TransGen Biotech, China). The gene expression was detected by qPCR with FastStart universal SYBR Green Master (Roche, United States) using QuantStudio 1 (Thermo Fisher, United States). GAPDH was used as an internal control. The target genes’ relative mRNA expression level was quantified with the 2^−ΔΔCT^ method. The primers used (Sangon Biotech, China) are shown in Table 1.

**TABLE 1:** Primers sequences used for qPCR.

Gene Name	Forward primer (5′to 3′)	Reverse primer (5′to 3′)
GAPDH	GAA​TGG​GCA​GCC​GTT​AGG​AA	AGG​AGA​AAT​CGG​GCC​AGC​TA
IL-1β	TGC​CAC​CTT​TTG​ACA​GTG​ATG	TGA​TGT​GCT​GCT​GCG​AGA​TT
IL-6	TGG​TCT​TCT​GGA​GTA​CCA​TAG​C	TGT​GAC​TCC​AGC​TTA​TCT​CTT​GG
TNF-α	GAG​GCC​AAG​CCC​TGG​TAT​G	CGG​GCC​GAT​TGA​TCT​CAG​C
TGF-β1	ACA​ATT​CCT​GGC​GTT​ACC​TT	AGC​CCT​GTA​TTC​CGT​CTC​C
collagen I	GTG​TTC​CCT​ACT​CAG​CCG​TC	ACT​CGA​ACG​GGA​ATC​CAT​CG
MMP9	AAC​CTC​CAA​CCT​CAC​GGA​C	CAG​CGT​GGT​GTT​CGA​ATG​G
E-cadherin	CAG​GTC​TCC​TCA​TGG​CTT​TGC	CTT​CCG​AAA​AGA​AGG​CTG​TCC
N-cadherin	AGG​CTT​CTG​GTG​AAA​TTG​CAT	GTC​CAC​CTT​GAA​ATC​TGC​TGG
α-SMA	AGCGGGCATCCACGAAAC	TTG​ATC​TTC​ATG​GTG​CTG​GGT

### 2.7 RNA sequencing analysis

Total RNA was extracted from the lung tissues using TRIzol^®^ Reagent according the manufacturer’s instructions (Magen). RNA samples were detected based on the A260/A280 absorbance ratio with a Nanodrop ND-2000 system (Thermo Scientific, United States), and the RIN of RNA was determined by an Agilent Bioanalyzer 4150 system (Agilent Technologies, CA, United States). Only qualified samples will be used for library construction. Paired-end libraries were prepared using a ABclonal mRNA-seq Lib Prep Kit (ABclonal, China) following the manufacturer’s instructions. The mRNA was purified from 1 μg total RNA using oligo (dT) magnetic beads followed by fragmentation carried out using divalent cations at elevated temperatures in ABclonal First Strand Synthesis Reaction Buffer. Subsequently, first-strand cDNAs were synthesized with random hexamer primers and Reverse Transcriptase (RNase H) using mRNA fragments as templates, followed by second-strand cDNA synthesis using DNA polymerase I, RNAseH, buffer, and dNTPs. The synthesized double stranded cDNA fragments were then adapterligated for preparation of the paired-end library. Adaptor-ligated cDNA were used for PCR amplification. PCR products were purified (AMPure XP system) and library quality was assessed on an Agilent Bioanalyzer 4150 system. Finally, the library preparations were sequenced on an Illumina Novaseq 6000 and 150 bp paired-end reads were generated. The data generated from Illumina platform were used for bioinformatics analysis. All of the analyses were performed using an in-house pipeline from Shanghai Applied Protein Technology. FeatureCounts (http://subread.sourceforge.net/) was used to count the reads numbers mapped to each gene. And then FPKM of each gene was calculated based on the length of the gene and reads count mapped to this gene. Differential expression analysis was performed using the DESeq2 (http://bioconductor.org/packages/release/bioc/html/DESeq2.html), DEGs with | log2FC | > 1 and Padj <0.05 were considered to be significantly different expressed genes.

### 2.8 Immunohistochemistry

The lung tissue sections underwent de-paraffinization and hydration, followed by antigen retrieval in 3% H_2_O_2_ for 10 min. Subsequently, the sections were incubated with goat serum and then exposed to α-SMA antibody (1:1500) or E-cadherin antibody (1: 500) at 4°C overnight. The sections were then incubated with goat anti-rabbit IgG, followed by incubation with 0.05% diaminobenzidine and restained with hematoxylin.

### 2.9 Western blot analysis

Total proteins from lung tissue and cells were extracted using RIPA lysis buffer (Thermo Fisher, United States) supplemented with a phosphatase and protease inhibitor cocktail. Then, the samples were lysed on ice for 20 min and centrifuged at 12,000 rpm for 10 min. The total protein concentrations in the supernatant were measured by the BCA protein kit (Thermo Fisher, United States). The proteins were separated by SDS-PAGE and transferred onto PVDF membranes (Millipore, United States). After blocking the membranes with 5% skimmed milk for 2 h, the membranes were sealed with different primary antibodies against TGF-β1, TGFBR2, α-SMA, E-cadherin, N-cadherin, collagen I, p-PI3K, PI3K, p-Akt1, Akt1, and β-actin at 4°C overnight. The membranes were then incubated for 2 h at room temperature with horseradish peroxidase (HRP)-coupled goat anti-mouse or goat anti-rabbit IgG secondary antibody. The protein bands were visualized using the ECL luminescence detection kit, and the grayscale values of the protein bands were analyzed using ImageJ software.

### 2.10 Statistical analysis

All data were presented as mean ± standard deviation (SD). GraphPad Prism 8.0 software was used to perform One-way ANOVA analysis and to graph. *p* < 0.05 was considered statistically significant.

## 3 Results

### 3.1 Tan attenuated bleomycin-induced pulmonary fibrosis in mice

**FIGURE 1:**
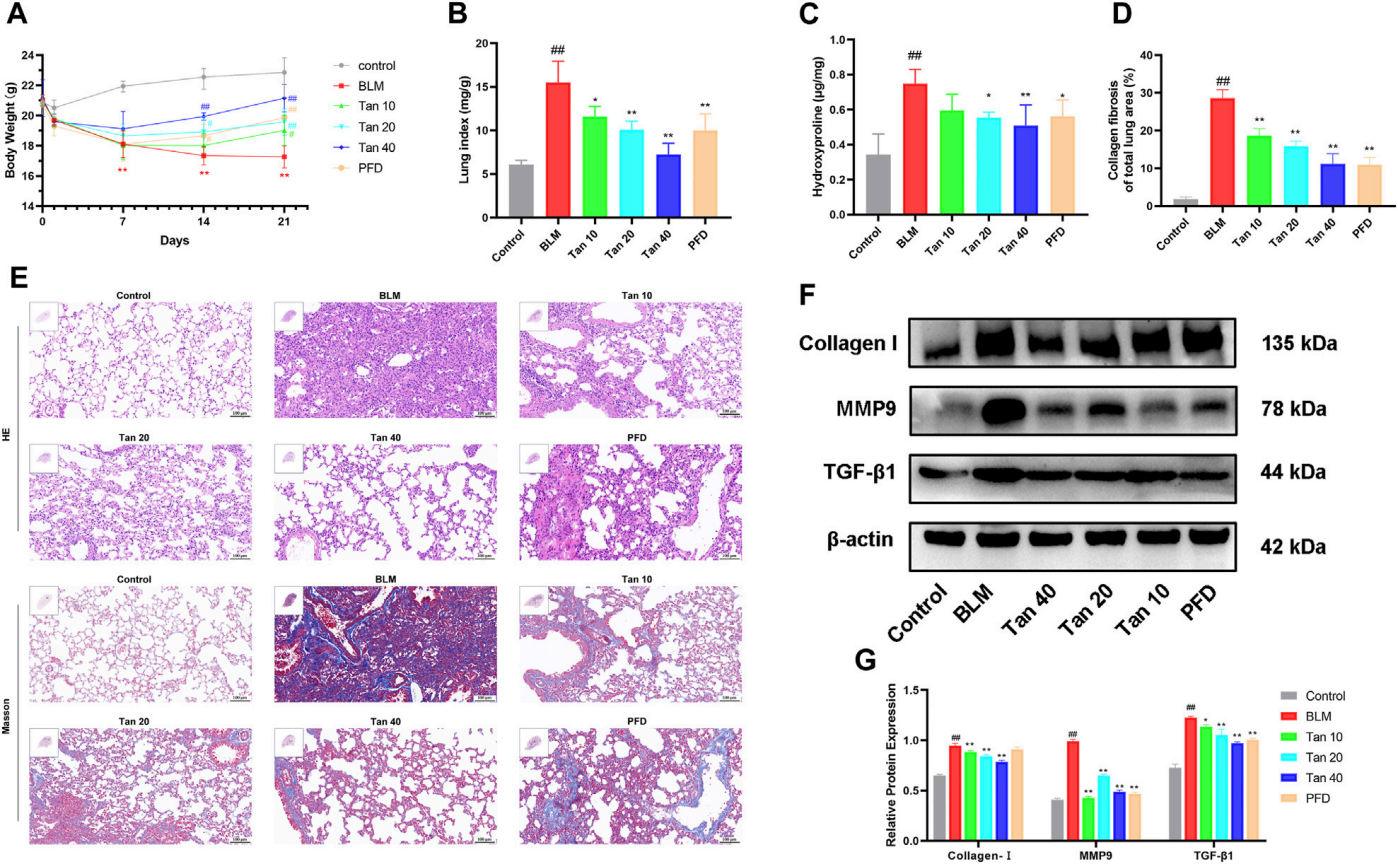
Tan attenuated BLM-induced pulmonary fibrosis in mice. **(A)** Body weight changes in various groups of mice at different time points (n = 6). **(B)** Lung index on day 21 (n = 6). **(C)** The hydroxyproline content in the mice lung tissues (n = 6). **(D)** Area statistics of blue collagen fibers in lung tissue as indicated in Materials and methods. **(E)** The images of H&E and Masson staining, scale bar, 100 µm. **(F)** Western blotting of collagen I, MMP9, and TGF-β1 proteins in mice lung. **(G)** Quantification of Western blot bands using ImageJ (n = 3). The data represent the mean ± SD. ^#^ *p* < 0.05, ^##^ *p* < 0.01, compared with the control group. *p < 0.05, **p < 0.01, compared with the model group.

To investigate the role of Tan in pulmonary fibrosis, a pulmonary fibrosis mouse model was established by tracheal drip injection of BLM. The results showed that the weight of BLM-treated mice decreased significantly, while Tan or PFD could increase the weight of the mice (Figure 1A). Meanwhile, Tan or PFD could effectively reduce the lung index and the hydroxyproline content of the lung tissue (Figures 1B,C). H&E and Masson staining results showed that, compared with the control group, the BLM group mice had severely damaged alveolar structures with massive inflammatory cell infiltration, alveolar wall thickening, and blue collagen deposition. These effects were significantly reduced by treatment with Tan or PFD (Figure 1E). Furthermore, Tan or PFD treatment reduced the Ashcroft score and the area of collagen fibers in the lung tissue of mice with pulmonary fibrosis (Figure 1D, Supplementary Figure S1A). Then, we measured the expression of collagen I, TGF-β1 and the core protein MMP9, markers of pulmonary fibrosis, by Western blotting. The data showed that BLM induced the expression of collagen I, TGF-β1, and MMP9, and Tan or PFD treatment significantly reduced this increase (Figures 1F,G). Consistent with the Western blotting results, qPCR results indicated that Tan intervention significantly reduced collagen I, TGF-β1, and MMP9 mRNA levels (Supplementary Figures S1B–D). Collectively, these results suggest that Tan could mitigate BLM-induced pulmonary fibrosis.

### 3.2 Tan reduced inflammation and oxidative stress in mice lung tissues

**FIGURE 2:**
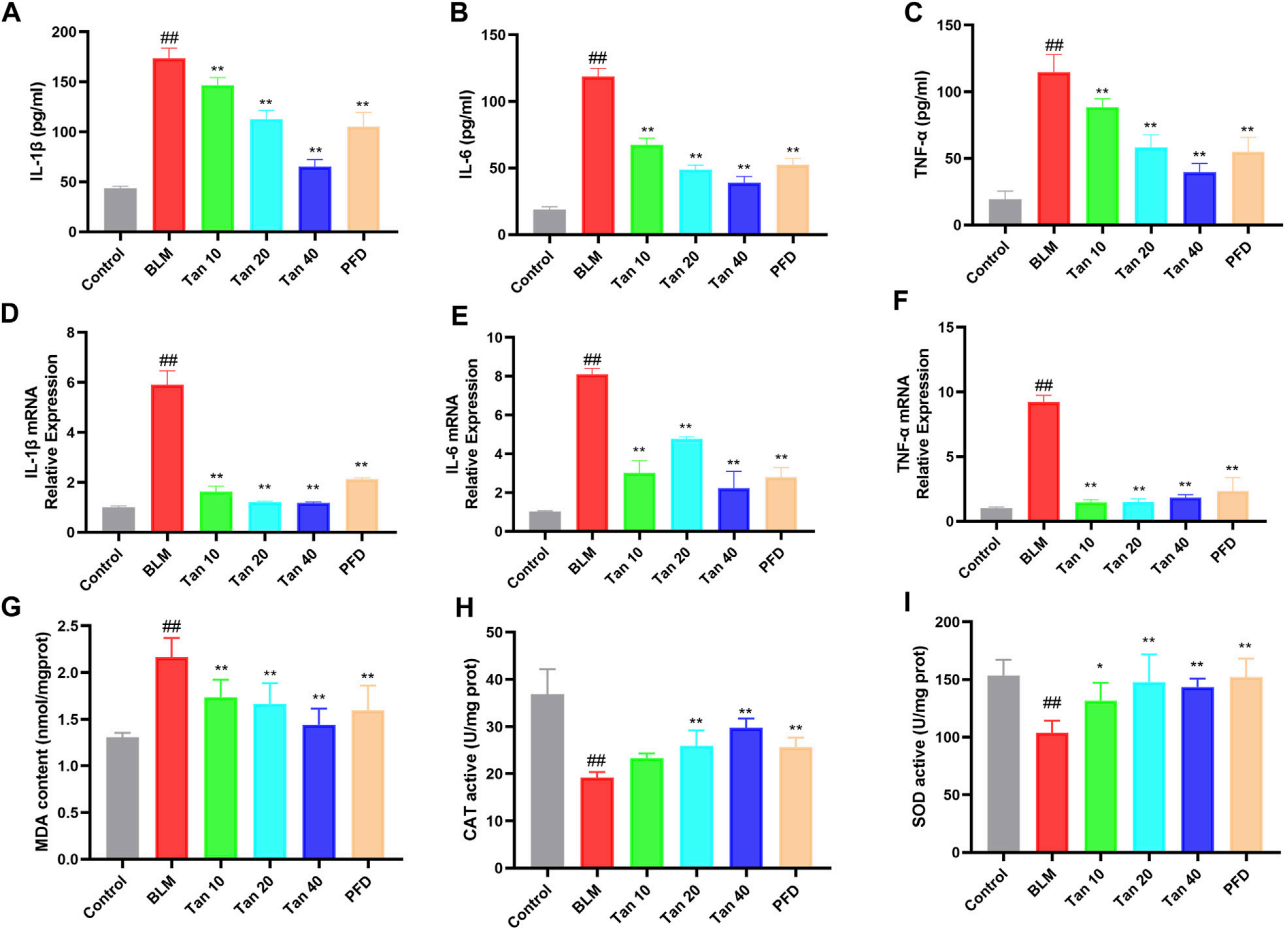
Tan reduced inflammation and oxidative stress in mice lung tissues. The secretion of IL-1β **(A)**, IL-6 **(B)**, and TNF-α **(C)** in lung tissue homogenates were measured by ELISA (n = 6). The mRNA levels of IL-1β **(D)**, IL-6 **(E)**, and TNF-α **(F)** in lung tissue were measured by qPCR (n = 3). The MDA content **(G)**, CAT **(H)**, and SOD **(I)** activity were determined in the mice lung tissues (n = 6). ^##^ *p* < 0.01, compared with the control group. *p < 0.05, **p < 0.01, compared with the model group.

Studies have found that inflammation and oxidative stress also play an important role in the development of pulmonary fibrosis (Cameli et al., 2020; Q; Zhang et al., 2023). Hence, to evaluate the anti-inflammatory and antioxidant effects of Tan, we measured inflammation and oxidative stress-related indicators in mice lung tissues. The secretion and mRNA expression of IL-1β, IL-6, and TNF-α were significantly higher in the lung tissue of BLM-treated mice compared to the control group, and Tan decreased them in a dose-dependent manner (Figures 2A–F). In addition, the Tan intervention significantly reduced MDA levels and increased CAT and SOD activities (Figures 2G–I).

### 3.3 Transcriptomic analysis of Tan treating pulmonary fibrosis

**FIGURE 3:**
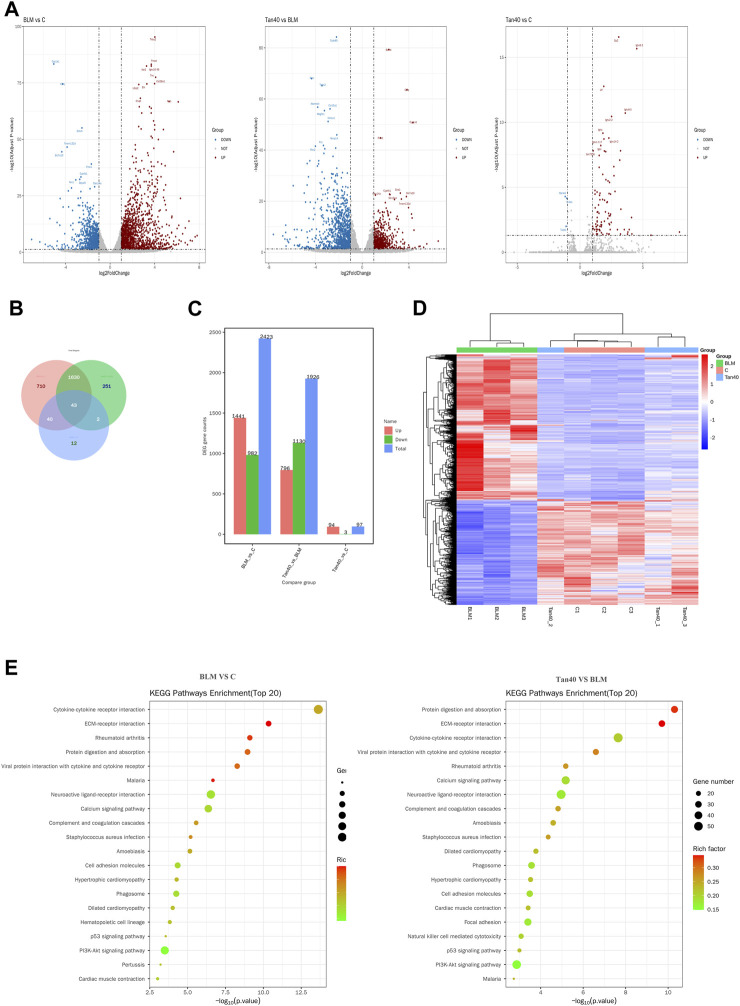
Transcriptomic effects of Tan on the lungs of mice with pulmonary fibrosis (n = 3). **(A)** Volcano plot of mRNA expression in each group of lung tissue. **(B)** Venn diagram of differentially expressed mRNAs between the three groups. **(C)** Number of DEGs (Differential Expressed Genes) in each group. **(D)** Heatmap showing the hierarchical clustering of mRNAs in the lung tissues of different groups. **(E)** The top 20 KEGG pathway enrichment analysis in control vs BLM, BLM vs Tan 40.

We used DEseq2 for differential analysis, with the screening being | log_2_FC | > 1 and Padj <0.05. Volcano plot showing changes in expression of different genes between the three groups (Figure 3A). Compared with the control group, a total of 2423 DEGs were identified in the BLM group, of which 1441 were upregulated and 982 were downregulated. After treatment with Tan, a total of 1926 DEGs were identified. Among them, 796 genes were upregulated, while 1130 genes were downregulated (Figure 3B). The Venn diagram showed 43 DEGs between the groups, which may be the critical DEG for the Tan treatment of pulmonary fibrosis (Figure 3C). As shown in Figure 3D, heat map clustering analysis was performed on the DEGs. In addition, the KEGG pathway enrichment analysis showed that Tan treatment of pulmonary fibrosis mice mainly affected ECM-receptor interaction, PI3K/Akt signaling pathway, P53 signaling pathway and other related pathways. The top 20 KEGG pathways were showed in Figure 3E.

### 3.4 Tan inhibited BLM-Induced epithelial-mesenchymal transition process *in Vivo*

**FIGURE 4:**
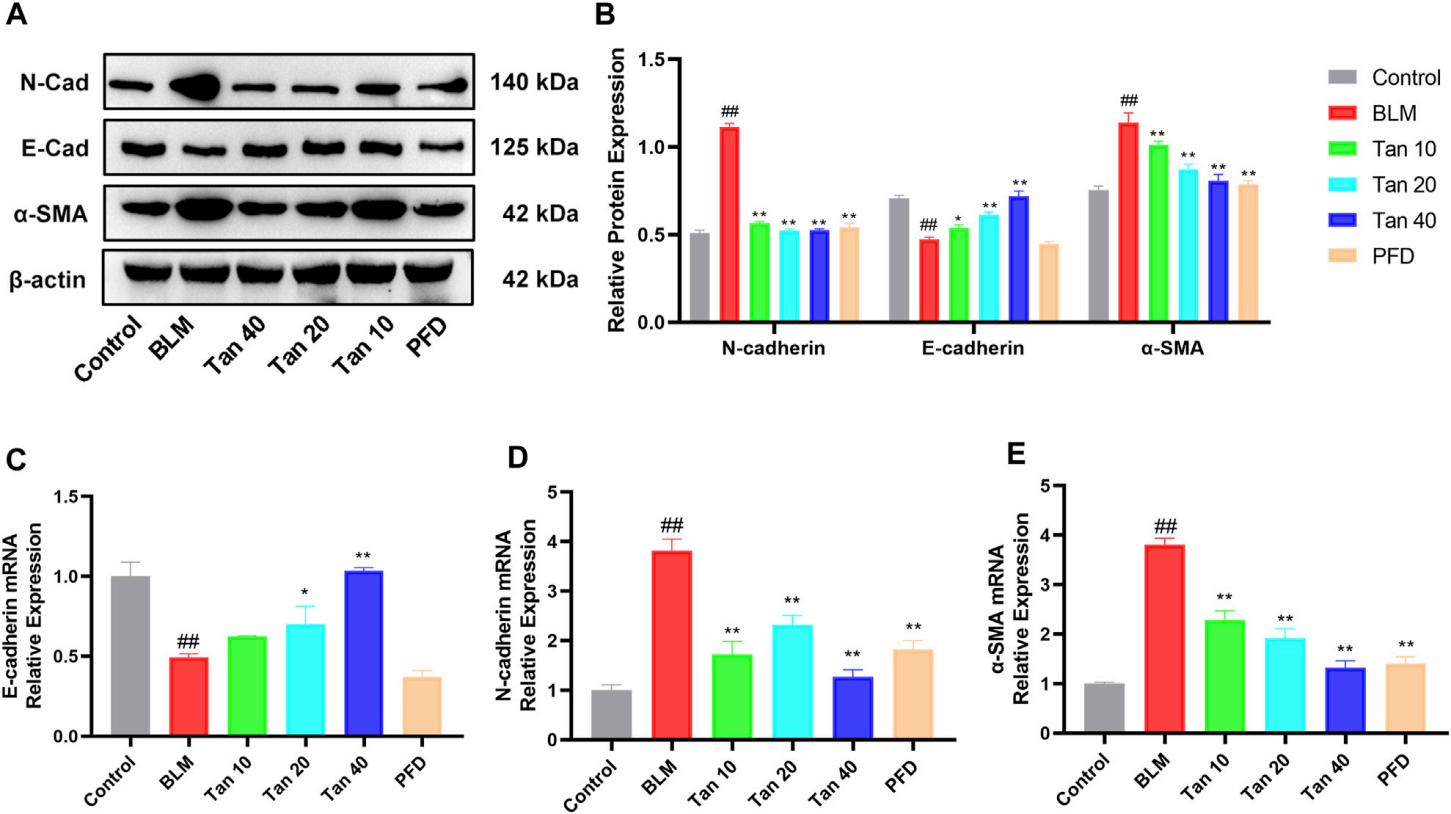
Tan reduced BLM-induced EMT in pulmonary fibrosis. **(A)** Western blotting of E-cadherin, N-cadherin, and α-SMA proteins in mice lung. **(B)** Grayscale analysis of target proteins using β-actin as an internal reference (n = 3). The mRNA levels of E-cadherin **(C)**, N-cadherin **(D)**, and α-SMA **(E)** in lung tissue were measured by qPCR (n = 3). ^##^ *p* < 0.01, compared with the control group. *p < 0.05, **p < 0.01, compared with the model group.

**FIGURE 5:**
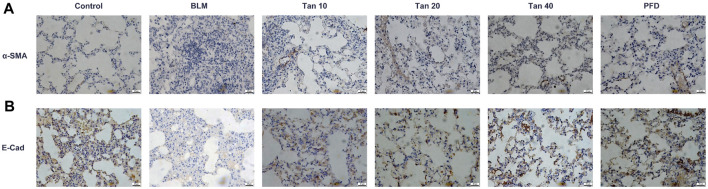
Immunohistochemical results of α-SMA **(A)** and E-cadherin **(B)** in lung tissue, scale bar, 40 µm.

EMT is a critical pathological process in pulmonary fibrosis, manifested mainly in the transformation of epithelial cells into fibroblasts, leading to a downregulation of the expression of the epithelial marker E-cadherin and an upregulation of the mesenchymal phenotypic markers N-cadherin and α-SMA (Rout-Pitt et al., 2018; Miao et al., 2022). As shown in Figures 4A,B, the expression of E-cadherin was reduced and the expression of N-cadherin and α-SMA was increased in the lung tissue of BLM-treated mice compared to the control group. The qPCR results were consistent with the Western blotting results, which showed that Tan intervention significantly upregulated E-cadherin gene expression and downregulated N-cadherin and α-SMA gene expression (Figures 4C–E). Immunohistochemical staining revealed a significant increase in a-SMA expression and decrease in E-cadherin expression in the BLM group. However, intervention with Tan reversed these changes (Figures 5A, B).

### 3.5 Tan alleviated TGF-β1-induced epithelial-mesenchymal transition process in A549 cells

**FIGURE 6:**
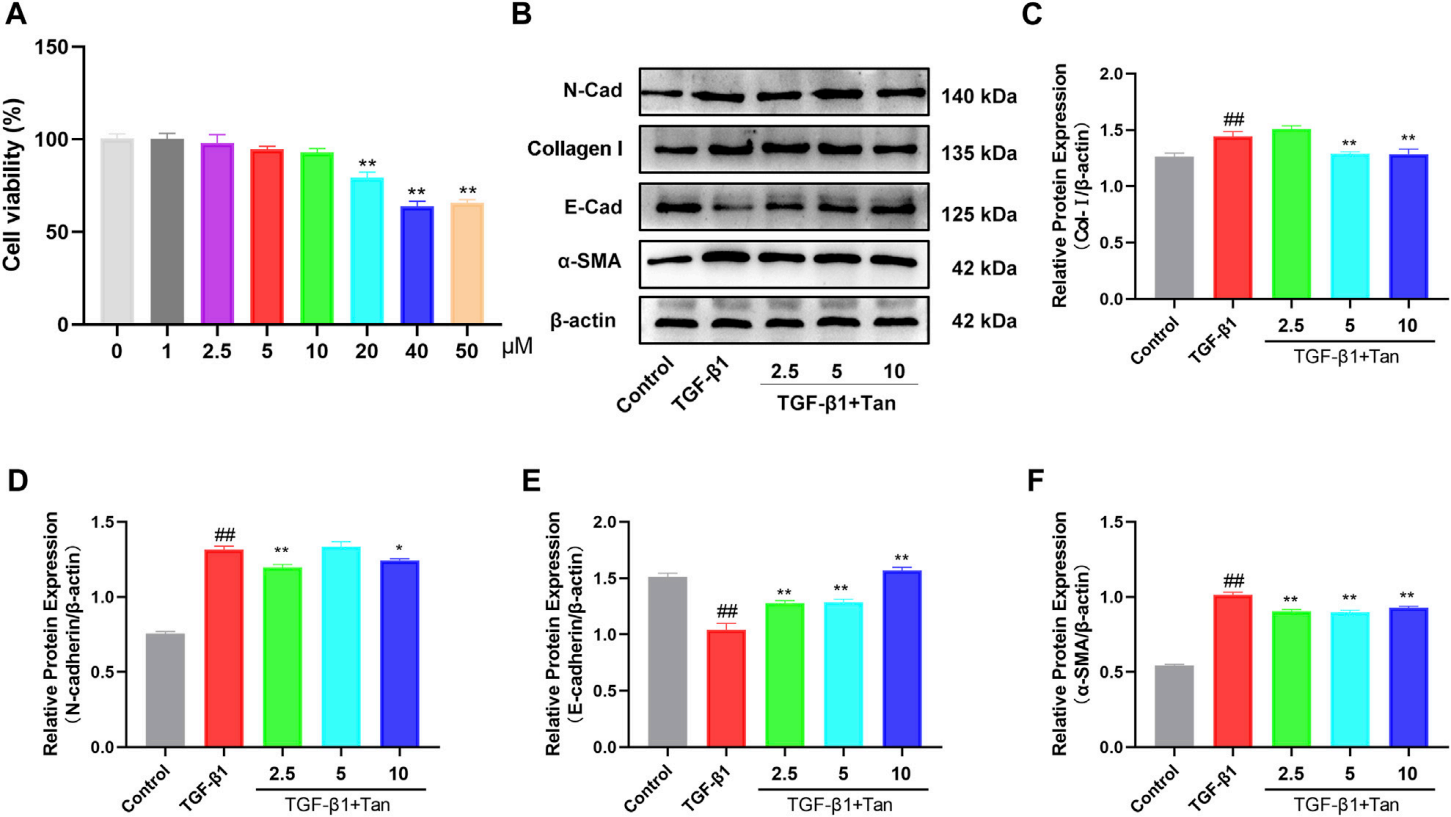
Tan alleviated the TGF-β1-induced EMT *in vitro*. **(A)** The effect of Tan on the viability of A549 cells. **(B)** Western blotting of collagen I, E-cadherin, N-cadherin, and α-SMA protein. **(C)** Quantification of the collagen I/β-actin ratio. **(D)** E-cadherin/β-actin ratio. **(E)** N-cadherin/β-actin ratio. **(F)** α-SMA/β-actin ratio. ^##^ *p* < 0.01, compared with the control group. *p < 0.05, **p < 0.01, compared with the model group.

To further elucidate the mechanism of Tan in pulmonary fibrosis, we examined the effect of Tan on TGF-β1-induced EMT in A549 cells. The CCK-8 results showed no cytotoxic effect of Tan on A549 cells in the concentration range of 0–10 μM (Figure 6A). Tan effectively reduced the upregulation of TGFBR2 induced by 5 ng/mL TGF-β1 stimulation (Supplementary Figure S2). The results presented in Figures 6B–F indicate a significant increase in the protein expression of collagen I, N-cadherin, and α-SMA, along with a decrease in E-cadherin expression in A549 cells following TGF-β1 stimulation. In contrast, Tan administration decreased the expression of collagen I, N-cadherin, and α-SMA and increased the expression of E-cadherin. In conclusion, Tan can inhibit TGF-β1 induced EMT process to alleviate pulmonary fibrosis.

### 3.6 Tan inhibited PI3K/Akt signaling pathway *in Vitro* and *in Vivo*

**FIGURE 7:**
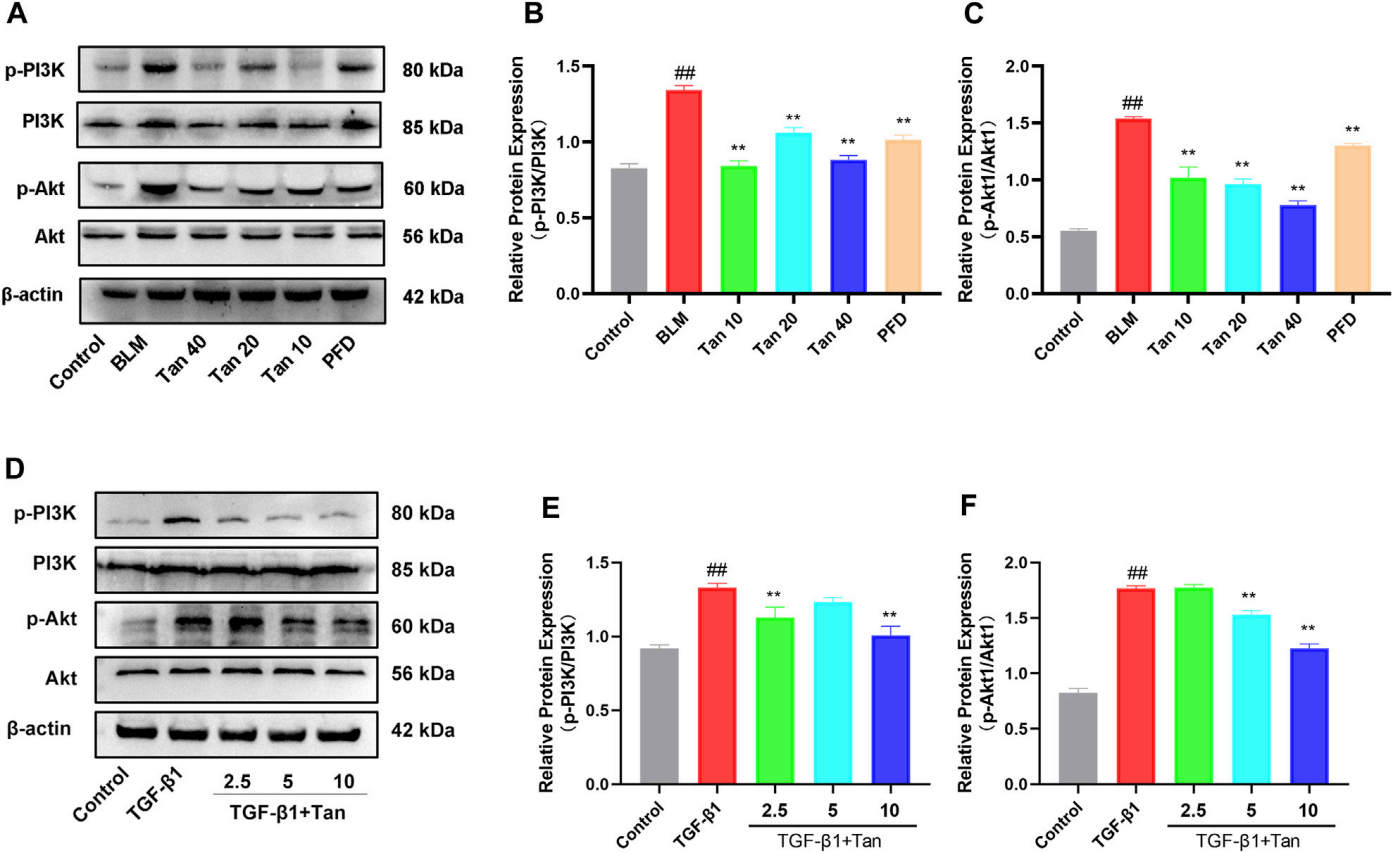
Tan inhibited the PI3K/Akt signaling pathway in pulmonary fibrosis. **(A)** Western blotting of p-PI3K, PI3K, p-Akt, and Akt proteins in mice lung. **(B)** Quantification of the p-PI3K/PI3K ratio. **(C)** p-Akt/Akt ratio. **(D)** Western blotting of p-PI3K, PI3K, p-Akt, and Akt proteins in TGF-β1-stimulated A549 cells. **(E)** p-PI3K/PI3K ratio. **(F)** p-Akt/Akt ratio. ^##^ *p* < 0.01, compared with the control group. *p < 0.05, **p < 0.01, compared with the model group.

Transcriptomic results showed that the PI3K/Akt signaling pathway was enriched with more DEGs. To investigate the potential relationship between the ameliorative effect of Tan on pulmonary fibrosis mice and the PI3K/Akt signaling pathway, we conducted Western blotting analysis to detect relevant proteins in this pathway. The results revealed a significant increase in the expression of p-PI3K and p-Akt1 in the lung tissues of pulmonary fibrosis mice. However, Tan treatment remarkably inhibited the expression of p-PI3K and p-Akt1 (Figures 7A–C). Furthermore, Tan also exhibited a suppressive effect on the expression of p-PI3K and p-Akt1 in TGF-β1-induced A549 cells (Figures 7D–F). These findings collectively suggest that Tan exerts its ameliorative effects on pulmonary fibrosis through the modulation of the PI3K/Akt signaling pathway.

## 4 Discussion

Pulmonary fibrosis represents the final stage of multiple acute and chronic lung ailments, culminating in respiratory failure and mortality. While pirfenidone and nintedanib have received FDA approval for pulmonary fibrosis treatment, they do not significantly reduce patient mortality (Trachalaki, Irfan, and Wells, 2021). Consequently, the quest for novel drugs to address pulmonary fibrosis is a pressing concern. Numerous studies have highlighted the antifibrotic advantages of the active constituents found in herbal medicines (Wang et al., 2021). Tan, an active constituent of the Chinese medicine Chen Pi, exhibits a diverse range of pharmacological activities (Cheng et al., 2023). Research has demonstrated its potential as an antiviral agent by hindering the entry of the SARS-CoV-2 virus into cells (H. Wang et al., 2023). In this study, we elucidated the protective mechanism of Tan against pulmonary fibrosis. Transcriptome analysis of lung tissue samples from mice with pulmonary fibrosis identified several relevant DEGs. The findings suggest that Tan’s amelioration of pulmonary fibrosis is associated with the PI3K/Akt signaling pathway. Further experimental validation showed that Tan inhibited TGF-β1-induced EMT in epithelial cells by suppressing PI3K/Akt signaling, which effectively attenuated pulmonary fibrosis.

BLM, an anti-tumor drug, has been utilized in animal experiments to induce pulmonary fibrosis due to its significant lung toxicity (Mouratis and Vassilis, 2011). After tracheal instillation of BLM, the lung epithelial cells undergo damage, resulting in a severe inflammatory response in the lungs. This response produces high levels of pro-inflammatory cytokines such as TNF-α, IL-1β, and IL-6, which in turn promote the recruitment of macrophages and lymphocytes, as well as the activation of fibroblasts (Xin et al., 2019; Lv et al., 2023). Our study found that the stimulation of BLM led to a significant increase in the secretion and expression of pro-inflammatory cytokines in lung tissue. However, the administration of Tan resulted in a significant reduction in the levels of these inflammatory factors. In addition, Tan was observed to alleviate the weight loss and elevated lung index caused by BLM in mice. Interestingly, the improvement observed with Tan treatment was more significant than that observed with pirfenidone treatment. The progression of pulmonary fibrosis is dependent on the proliferation and differentiation of fibroblasts. Myofibroblasts, which overexpress α-SMA, play a crucial role in this process by secreting Collagen I and Collagen III. This leads to an increase in extracellular matrix (ECM) deposition in the interstitial lung matrix, resulting in changes in matrix composition and increased lung tissue stiffness (X. Liu et al., 2018). The enzyme MMP9 is responsible for breaking down the extracellular matrix (ECM) and changing the balance between ECM and interstitial collagen, which can lead to the formation of fibrosis (G. Li et al., 2019a). The pathological histology analysis revealed severe damage to lung tissue, along with increased collagen deposition and higher hydroxyproline content in the mice with pulmonary fibrosis, which aligns with previous findings (Saito et al., 2017). However, Tan’s intervention showed a significant improvement in lung histopathological damage and collagen deposition in the mice. Additionally, the study found that Tan reduced the expression of Collagen I, MMP9, and TGF-β1 in BLM-treated mice, which are all indicators of pulmonary fibrosis.

The role of oxidative stress in the development of pulmonary fibrosis is well established (Park et al., 2021). Studies have shown that exposure to BLM can lead to DNA damage, generation of reactive oxygen species, and a decrease in serum T-SOD, CAT, and GSH activities, while increasing MDA expression (Mouratis and Vassilis, 2011). Inhibition of oxidative stress has been shown to improve the pulmonary fibrosis process in mice. Our study found that compared to the control group, mice with pulmonary fibrosis treated with BLM had significantly higher MDA levels and lower SOD and CAT activity in lung tissue. However, treatment with Tan significantly inhibited BLM-induced oxidative stress in the lung tissue of mice.

In addition to resident lung fibroblasts, EMT is believed to be the primary source of myofibroblasts in pulmonary fibrosis (P. Wang, Yan, et al., 2022). Studies have shown that about one-third of fibroblasts in pulmonary fibrosis originate from epithelial cells (Tanjore et al., 2009). These cells undergo structural and functional changes to become mesenchymal cells when exposed to oxidative stress or TGF-β1, leading to the development of EMT (Rout-Pitt et al., 2018). As a result, we examined the impact of Tan on EMT in our study. The study found that Tan increased the levels of protein and gene of E-cadherin and decreased the levels of protein and gene of N-cadherin and α-SMA. A549 cells were used to induce the phenotypic transformation caused by TGF-β1 and Western blotting was employed to analyze the effect of Tan on the epithelial-mesenchymal transition (EMT) phenotype The results indicated that consistent with previous studies, E-cadherin levels decreased and collagen I, N-cadherin, and α-SMA expression increased after TGF-β1 treatment, but improved after treatment with Tan(Q. Chen et al., 2023). Therefore, the research demonstrated that Tan can suppress EMT and inhibit pulmonary fibrosis.

The process of TGF-β1-induced EMT involves the activation of non-Smad-dependent signaling pathways such as PI3K/Akt, MAPK, RhoA, and NF-κB(Y.E. Zhang, 2017). PI3K/Akt signaling pathway has been identified as a crucial regulator of pulmonary fibrosis (J. Wang, et al., 2022). Studies have shown that inhibition of PI3K/Akt disrupts EMT and the use of Akt inhibitors can partially reverse EMT (Lin et al., 2014). TGF-β1 activates PI3K through the TGF-β receptor or EGF receptor. As a secondary messenger, PI3K activation causes the p110 subunit to bind with the p85 subunit, leading to the phosphorylation of the substrate PIP2. This conversion results in the formation of PIP3, which then binds to the PH structural domain of Akt, leading to its activation through phosphorylation (Hers et al., 2011; Saito et al., 2017). Activation of Akt can induce the expression of EMT-inducible transcription factors and phosphorylate Snail1 by inhibiting GSK-3β or activating NF-κB to promote EMT in squamous cell carcinoma cells (L. Zhang et al., 2013; Kaufhold and Bonavida, 2014). Moreover, sustained PI3K activation can aggravate BLM-induced pulmonary fibrosis by promoting the release of pro-inflammatory and pro-fibrotic factors (Kral et al., 2016). In conjunction with KEGG pathway enrichment analysis, we evaluated the expression of the PI3K/Akt pathway using Western blotting. The data show that Tan inhibits PI3K and Akt phosphorylation both *in vivo* and *in vitro*. Tan, a selective PI3K inhibitor, shows potential as a drug for treating upper respiratory tract infections (S. Chen et al., 2022). Furthermore, the inclusion of Tan in the diet can enhance the production of short-chain fatty acids. These fatty acids play a role in inhibiting the epithelial-mesenchymal transition (EMT) process by suppressing the PI3K/Akt/mTOR signaling cascade (B. Chen et al., 2021; D; Chen et al., 2020).Therefore, Tan may directly or indirectly act on PI3K, inhibiting the activation of the PI3K/Akt signaling pathway, and subsequently inhibiting the epithelial-mesenchymal transition (EMT) process in pulmonary fibrosis.

## 5 Conclusion

To conclude, this study provides evidence that Tan can improve pulmonary fibrosis both *in vivo* and *in vitro* by inhibiting EMT through the PI3K/Akt signaling pathway. The findings suggest that Tan could be a viable drug candidate for the treatment of pulmonary fibrosis.

## Data Availability

The datasets presented in this study can be found in online repositories. The names of the repository/repositories and accession number(s) can be found below: Sequence Read Archive (SRA)/PRJNA987041.

## Supplementary material

SUPPLEMENTARY FIGURE S1 Tan improved lung tissue scores and reduced mRNA expression of pulmonary fibrosis markers in mice with pulmonary fibrosis. **(A)** Ashcroft score. The mRNA expression of collagen I **(B)**, MMP9 **(C)** and TGF-β1 **(D)** was detected by qPCR in the lung tissue in each group of mice. Data represent the mean ± SD. ^#^ *P* < 0.05, ^##^ *P* < 0.01, compared with the control group. **P* < 0.05, ***P* < 0.01, compared with the model group.

SUPPLEMENTARY FIGURE S2 Tan treatment resulted in a decrease in the expression of the TGF-β1 receptor, TGFBR2. **(A)** Representative western blotting images showing the expression of TGFBR2. **(B)** Quantification of TGFBR2/β-actin ratio. ^##^ *P* < 0.01, compared with the control group. ***P* < 0.01, compared with the model group.
