# Supplementary material for: Tangeretin attenuates bleomycin-induced pulmonary fibrosis by inhibiting epithelial-mesenchymal transition via the PI3K/Akt pathway
Source: Front Pharmacol. 2023 Sep 15;14:1247800. doi: 10.3389/fphar.2023.1247800 (PMC10540689; doi:10.3389/fphar.2023.1247800)
Supplement: Supplementary file 1 [file Table1.docx]

Supplementary Material

Tangeretin attenuates bleomycin-induced pulmonary fibrosis by inhibiting Epithelial-Mesenchymal Transition via the PI3K/Akt pathway

Jiang Li^1,^ ^†^, Qian Wei^2,^ ^†^, Ke Song^1^,Youxin Wang^1^, Yuxin Yang^1^, Miao Li^1^, Jiaying Yu^1^,Guangxu Su^1^, Luyuan Peng^1^, Bendong Fu^1^, Pengfei Yi^1*^

*** Correspondence:** Corresponding Author: [yipengfei@jlu.edu.cn](mailto:yipengfei@jlu.edu.cn)

# Supplementary Figures and Tables

## Supplementary Figures


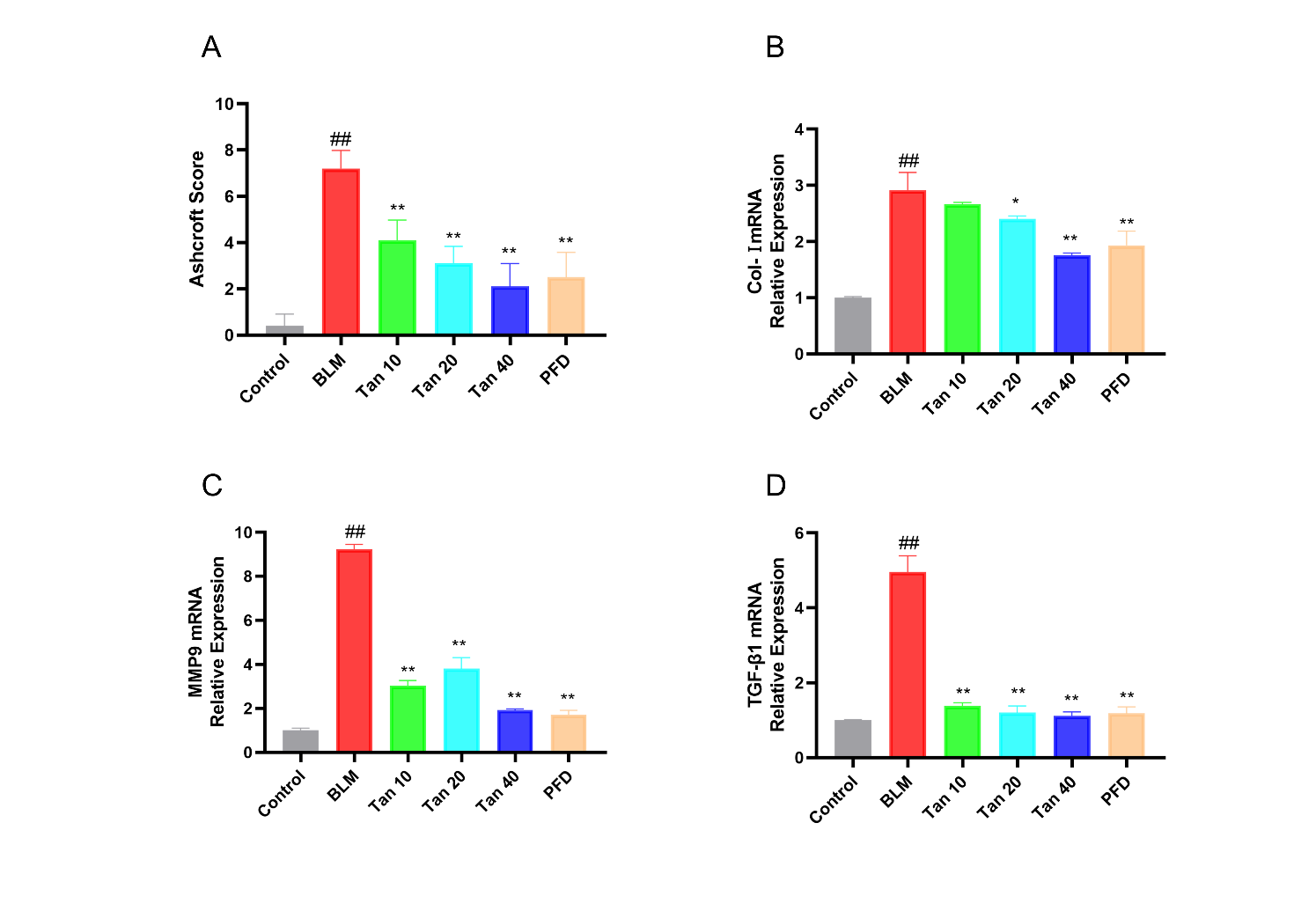


Supplementary Figure 1 Tan improved lung tissue scores and reduced mRNA expression of pulmonary fibrosis markers in mice with pulmonary fibrosis. (A) Ashcroft score. The mRNA expression of collagen I (B), MMP9 (C) and TGF-β1 (D) was detected by qPCR in the lung tissue in each group of mice. Data represent the mean ± SD. ^#^*P* < 0.05, ^##^*P* < 0.01, compared with the control group. **P* < 0.05, ***P* < 0.01, compared with the model group.


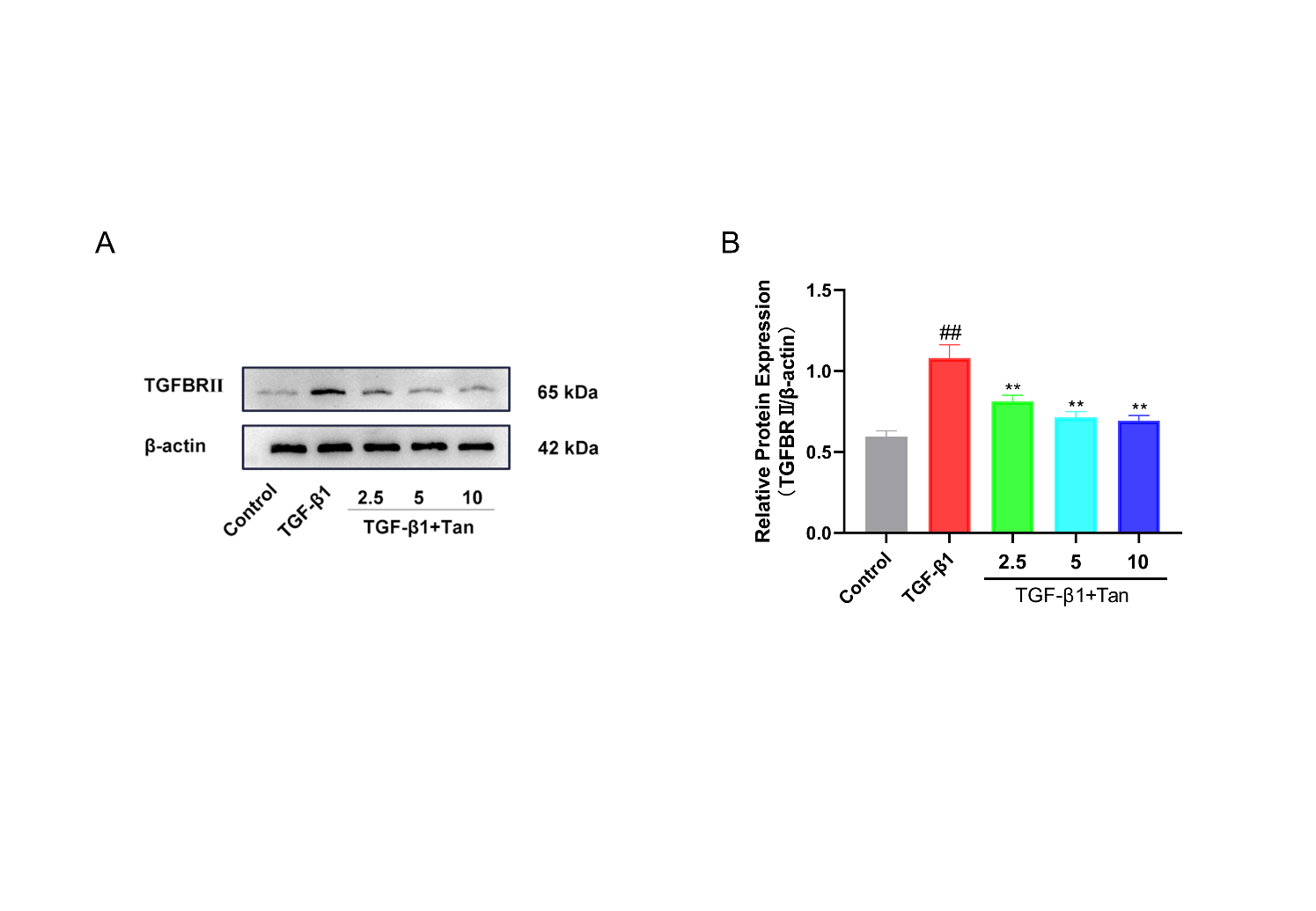


Supplementary Figure 2 Tan treatment resulted in a decrease in the expression of the TGF-β1 receptor, TGFBR2. (A) Representative western blotting images showing the expression of TGFBR2. (B) Quantification of TGFBR2/β-actin ratio. ^##^*P* < 0.01, compared with the control group. ***P* < 0.01, compared with the model group.
